# Host–Guest Engineering of MOF-808 for Random Lasing and Solid-State Emission

**DOI:** 10.1021/acsanm.5c02396

**Published:** 2025-07-18

**Authors:** Giuseppe Ficarra, Ashim Pramanik, Ludovico G. Barbata, Marco Cannas, Romy L. Ettlinger, Russel E. Morris, Gianpiero Buscarino, Fabrizio Messina, Alice Sciortino

**Affiliations:** † Physics and Chemistry Department - Emilio Segrè, University of Palermo, Via Archirafi 36, 90123 Palermo, Italy; ‡ INST Consortium for Materials Science and Technology, Via Giusti 9, 50125 Firenze, Italy; § EastChem School of Chemistry, University of St Andrews, North Haugh, St Andrews KY16 9ST, U.K.; ∥ TUM School of Natural Sciences, Technical University of Munich, Lichtenbergstr. 4, Garching bei München 85748, Germany

**Keywords:** metal–organic frameworks, random lasing, host−guest nanosystems, nanocomposites, portable lasing device

## Abstract

Fluorescent organic dyes have a broad range of applications across various fields. However, their use is threatened by stability issues such as photobleaching and aggregation-caused quenching that prevent them from showing solid-state luminescence and being used in high-power photonics applications for a long period. One possibility to overcome these problems is to embed dye molecules within a hosting platform. Metal–organic frameworks (MOFs) are among the best candidates to overcome these problems due to their porous nature, which provides excellent sorption capacities while ensuring stability for potential guest molecules, even in extreme environments. In this work, we investigate the optical performance of rhodamine B and coumarin 343 when interacting with Zr-based MOF-808. On one hand, we demonstrate that inclusion of dye molecules in MOF-808 cavities prevents aggregation-induced quenching, enabling the use of dyes in powdered form and enhancing their emission in solid-state applications, such as fingerprint detection. On the other hand, the dye–MOF interaction in solution reveals that MOF-808 nanoparticles act as efficient scatterers, significantly enhancing random lasing emission by narrowing the emission spectra and reducing the lasing threshold. The lasing performance is shown to be dependent on the MOF concentration and excitation intensity, with an optimal concentration minimizing the threshold and bandwidth. Finally, we demonstrate the feasibility of combining MOF-808 nanoparticles and dyes into polymeric thin films, where the MOFs contribute to halving the lasing threshold, making the system suitable for portable lasing applications.

## Graphical abstract

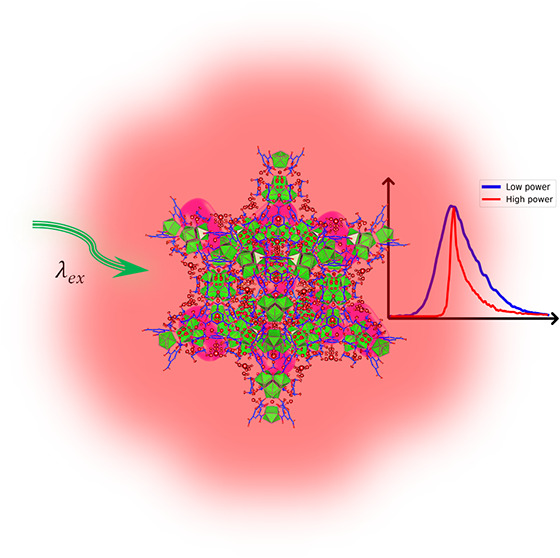

## Introduction

Fluorescent organic dyes are widely applied in various fields, including biomedical imaging, pigment and pharmaceutical industries, dye-sensitized solar cells, light harvesting, organic light-emitting devices (OLEDs), and optoelectronic devices.^1–3^ The interest in developing novel dyes has steadily grown over the years. However, when implementing these chromophores in the fabrication of optoelectronic devices, their excellent performances are often negatively affected due to stability issues.^4^

It is known that fluorescent dyes in solution, especially when high excitation irradiance is required, suffer from photobleaching phenomena, which cause an irreversible loss of fluorescence, limiting their possible applications in several optical and photonics fields. For example, the photobleaching restricts the single molecule detection as well as all fluorescence spectroscopy applications where high sensitivity is required.^5–7^ Photobleaching is also detrimental in systems in which such chromophores are used as the optical gain material; for example, a nematic liquid crystal droplet containing fluorescent dyes was reported to lase for only 10 min under a repetition rate of 100 Hz and a peak illumination power density of 10 MW/cm^2^.^4^ Improving the UV and thermal stability of dyes is also found to be a critical issue in the field of dye-sensitized solar cells (DSSCs), in which the performance of the cells is strictly related to the dye used as a sensitizer.^8^ Abdou and co-workers reported an ∼10-fold decrease in the optical density of coumarin dye solution after 1 h of UV exposure, clearly demonstrating the photobleaching effect.^4^ Moreover, several dyes suffer from the aggregation-caused quenching (ACQ) phenomenon that significantly reduces or completely disables their luminescence when they aggregate, rather than remaining well-separated as they usually do in a well-dispersed solution.^9^ When the dye concentration is increased, strong intermolecular π–π interactions are favored, allowing for an ordered stacking of the chromophores in the form of a cluster. These π–π interactions enable the formation of excimers between adjacent molecules, in which the excited species interact with the unexcited ones, adding multiple non-radiative pathways through which the excitation energy is more likely to be dissipated.^10^ This phenomenon prevents the full exploitation of organic dyes, especially in their powdered form as well as at high concentrations, in various fields such as fluorescence imaging (e.g., dactyloscopy) and photonics (e.g., lasing applications).^7^ A possibility to overcome these problems is to keep dye molecules separate by encapsulating them inside a matrix.^11^ Metal–organic frameworks (MOFs) are porous matrices that allow dye molecules to be adsorbed inside the pores.^12^ MOFs are nanohybrid materials composed of organic linkers and inorganic (metal–oxo clusters) building blocks, which self-assemble into 3D porous, crystalline frameworks.^13^ By appropriately selecting the building blocks, one can customize frameworks that feature cavities with a well-defined size (ranging from 0.2 to 3 nm) depending on the luminescent guest (LG) to be embedded.^14^ Owing to their high internal surface area values, MOFs can possess extraordinary adhesive properties, which highly facilitate postsynthetic impregnation with LGs. Indeed, most of these methods are based on liquid impregnation or gas-phase infiltration. Exploiting a MOF platform to host dye molecules reduces ACQ,^9^ as shown by Chen and co-workers with the composite system rhodamine B-bio-MOF-1, which yielded luminescence also in the solid state.^15^ Moreover, a trapped LG is affected by caging effects imposed by the host cavity, resulting in an increased luminescence lifetime (τ) and quantum yield (Φ).^16^ A MOF with a specific emission can also serve as a platform to sensitize the luminescence of the LG through fluorescence resonance energy transfer (FRET).^17^ Whenever the aim is to focus solely on the luminescent properties of the dye, one can either rely on a non-luminescent MOF or choose a dye with an emission that results in a significant red shift relative to the MOF.^18^ Additionally, a guest molecule confined within the pore may experience environmental effects altering the electric field it experiences, potentially leading to changes in its excited-state levels.^19^ Regarding the shielding properties of MOFs, Tuninetti and co-workers showed that a photoprotection effect is registered when photolabile biologically relevant molecules, such as folic acid (PteGlu), are trapped within the cavities of the framework ZIF-8, with a remarkable increase in stability toward irradiation with UV-A sources (up to 80% increase) compared to bare PteGlu.^20^ Similarly, in our previous work, we demonstrated that the inclusion of rhodamine B (RhB) molecules with the MOF-808 matrix allows reducing the photobleaching rate caused by UV radiation by a factor of 10.^21^ Photobleaching protection is compulsory for any photonics applications, such as a laser. In this regard, random lasing (RL) could be a particularly appropriate application of LG-impregnated MOFs, considering the photobleaching protection and the size of MOF particles, which can act as scatterers. RLs have gained significant attention over the years as a class of mirrorless laser-like sources with huge potential for photonic devices because of their low cost, tunability, and possibility to adjust the degree of spatial coherence of the output beam.^22,23^ RLs are typically composed of a photonic gain medium obtained by combining fluorescent gain molecules with passive scatterers.^24^ Unlike a conventional laser, where photons are retained within the resonant cavity by means of mirrors, in an RL, the feedback is provided by scatterers.^25^ In some RLs, the same nano-objects can play the role of both scatterers and gain media.^26^ If pumped over a certain energy threshold, at which gains exceed losses, the disordered active medium can yield an omnidirectional laser-like emission driven by light amplification along random paths created by multiple scattering events.^27^ Indeed, unidirectional laser emission is not expected, as the feedback mechanism is provided by random scattering events. Depending on the nature of the scattering events within the gain media, RLs are classified into two subgroups: incoherent and coherent RLs. Incoherent RLs originate from a series of non-resonant scattering events that take place before the photons leave the medium: in this case, the associated mean scattering length (*l*_s_), which occurs between two scattering events, is much bigger with respect to the emission wavelength (λ). Coherent RLs can originate from at least two different situations: systems characterized by efficient scattering (*l*_s_ *< λ*), which allows the formation of closed loops that act as resonant microcavities, or systems that act as very long light-path waveguides, in which it is possible to observe light amplification even through a non-resonant mechanism.^22^ Both the incoherent and coherent types of RLs are appealing for multiple applications. The low spatial coherence of incoherent random lasing can be exploited for speckle-free laser imaging, while coherent RLs are more suitable for security (such as random number generation or photonic barcodes) or super-resolution spectroscopy.^28,29^

Indeed, MOFs have already been proposed for photonic applications such as micro/nanolasers.^30^ Specifically, some works have reported that MOF nanoparticles could act as effective scattering centers when coupled to a gain medium.^31^ Although estimating their effective refractive index is challenging due to their porous structure, literature findings supported by theoretical calculations suggest values close to 1.4 for zirconium-based MOFs.^32^ Introducing MOF nanoparticles into an active medium may induce variations in the refractive index, thereby increasing the likelihood of scattering phenomena.

In the design of an RL, MOFs can also allow the spatial separation of the trapped LGs, minimizing the intramolecular energy loss and providing sufficient gain for the RL.^33^ In fact, MOFs can host sufficient gain media to achieve light amplification while suppressing non-radiative pathways. This strategy leads to the design of solid-state RLs with a low pump threshold.^34^ Xu and co-workers reported evidence of lasing phenomena arising from a solid-state system composed of bio-MOF-100 loaded with LDS 722 dye. Specifically, upon increasing the pump fluence above a certain threshold (0.67 mJ/cm^2^), the photoluminescence intensity resulted in dramatically amplified and sharp peaks that began to emerge.^32^ Liu and co-workers were able to combine LDS 798 (a cationic near-infrared dye) within the cavities of rho-ZMOF, showing evidence of lasing peaks arising above an ultralow threshold of 13.44 μJ/cm^2^.^35^ While previous literature clearly highlights how MOFs can be useful in the miniaturization of lasers, these studies focus on a single dye used as the gain medium, making it difficult to compare the effects of encapsulating a cationic dye versus neutral or anionic one. Additionally, the difference between amplified spontaneous emission and lasing can be uniquely identified only through time-resolved spectroscopic analysis, which has been hardly reported in previous works. Understanding these fundamental aspects can accelerate the development of novel portable devices.^30^

In this work, we explored the optical performance of two paradigmatic dyes, namely, RhB and coumarin 343 (Cou), when embedded within the cavities of MOF-808.^36^

The selection of MOF-808 as the hosting platform stems from its large cavities (18 Å diameter), which are well-suited for the encapsulation of chromophores, alongside the advantage that this MOF can be synthesized relatively easily under ambient conditions (a scheme of the precursors for the synthesis is shown in Figure 1a). Both RhB and Cou dyes are well-known for their good solubility, high values of quantum yield, and, most importantly, their molecular size, making them excellent candidates for fitting within the MOF’s pores.^37–39^ Considering the optical properties of these two dyes, it is possible to probe the optical response of these dyes–MOF nanosystems in the entire visible range.^39^ We embedded the dyes in MOF-808, resulting in two novel dual-emitting powdered systems. We showed, for the same system, that it is possible to trigger lasing phenomena above a certain energy threshold (the concept of the solid-state emitting systems is presented in Figure 1b). Moreover, we showed, as a proof of concept, that it is possible to exploit LG-MOFs for fingerprint detection thanks to their astonishing adhesive properties.^40^ Additionally, we investigated the potential role of MOF nanoparticles as scatterers in different gain media, considering that the absorption spectrum of MOF-808 is mostly dominated by scattering in the VIS range (a scheme of the liquid-phase system is reported in Figure 1c).^32^ We considered two different liquid gain media: an aqueous solution of RhB and a solution of Cou dissolved in ethanol. We progressively increased the quantity of MOF nanoparticles to determine the optimal concentration of scatterers, considering the associated energy threshold and narrowing of the lasing peak. We performed a nanosecond time-resolved analysis on the same systems, which allowed us to compare the decay kinetics associated with the lasing peaks to those related to the spontaneous emission of the bare dyes. We found evidence of a shortening of the decay kinetics whenever the pumping power exceeds a certain value, which demonstrates again the lasing threshold.^41^ Finally, we prepared a portable thin-film device from an aqueous solution of RhB and the biocompatible by poly­(vinyl alcohol) (PVA) to which MOF-808 nanoparticles were added as scatterers (concept in Figure 1d).^42,43^

**Figure 1:**
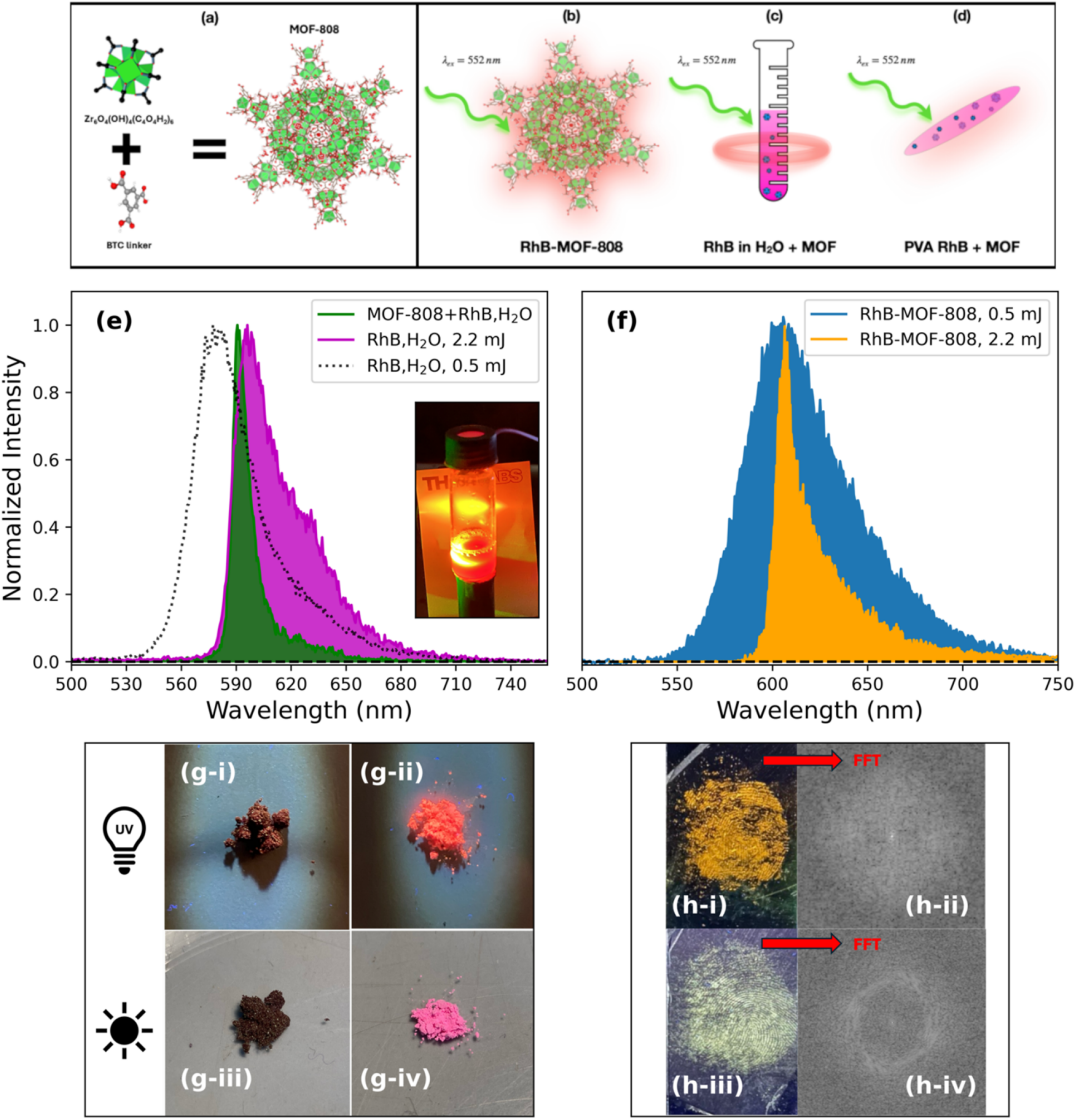
(a) Precursors used in the synthesis of MOF-808. (b) Scheme of the solid-state emitting system with RhB encapsulated within the pores of the MOF (RhB–MOF-808). (c) Scheme of the liquid-phase system in which MOF nanoparticles are used as passive scatterers (RhB in H_2_O + MOF). (d) Scheme of the thin-film system in which MOF nanoparticles are used as passive scatterers (PVA RhB film + MOF). (e) Fluorescence emission of bare RhB in water pumped at 2.2 mJ (magenta filled curve) compared with the emission of bare RhB at 0.5 mJ (black dotted curve) and lasing emission of a solution of RhB in water with the addition of MOF nanoparticles as scatterers (relative picture in the inset). (f) Photoluminescence of powdered RhB embedded in MOF-808 (blue filled curve) and lasing arising from the same powder (orange filled curve). (g-i) Bare RhB under UV light. (g-ii) RhB–MOF-808 under UV light. (g-iii) Bare RhB under daylight (g-iv) RhB–MOF-808 exposed to daylight. (h) Impression of a fingerprint on (h-i) bare Cou dye and (h-iii) Cou–MOF-808 powder. (h-ii) FFT transform of a portion of the fingerprint impressed on bare Cou. (h-iv) FFT transform of the same portion of the fingerprint as that obtained with Cou–MOF-808 powder.

We observed the onset of RL even in the thin film prepared without the addition of scattering centers. This finding is consistent with previous reports, which attribute the phenomenon either to the amplification of specific photonic modes within the disordered gain medium or to the formation of random cavities, arising from refractive index inhomogeneities, where the narrow emission peaks correspond to actual laser cavity modes. Nevertheless, we showed that the incorporation of MOF nanoparticles leads to a significant decrease in the lasing threshold compared to the case of the bare RhB film.^43,44^

The results are very promising in view of the prospective use of LG-MOFs as a versatile platform for multiple photonic applications and reveal important details about the fundamental mechanisms controlling the light emission from optical dyes embedded in MOFs.

## Experimental Methods

### Preparation of Samples

MOF-808 nanoparticles were synthesized following a room-temperature (25 °C) protocol previously reported by Dai and co-workers.^45^ 1.2 mg of Zr6-oxo clusters have been mixed in a mixture of 5 mL of water and 3 mL of formic acid under continuous stirring at 600 rpm. Then, 5 mL of water was added, and the solution was stirred until it turned transparent. Thereafter, 150 mg (0.7 mmol) of 1,3,5-benzenetricarboxylic acid was added, and the reaction mixture was left to stir overnight. The resulting suspension was centrifuged at 14,500 rpm for 15 min to separate the solid part, which was washed several times with 40 mL of water and 80 mL of ethanol. Finally, the solid was dried in an oven at 60 °C for 3 h. The XRD pattern (Figure S1b) showed several sharp peaks indicating a highly crystalline structure already discussed by Xuan and co-workers.^46^ SEM analysis of MOF-808 powders (Figure S1a) suggests the presence of monodispersed nanoparticles and allows one to estimate a size distribution with a mean value of 150 nm, which results in agreement with previous literature studies. To embed rhodamine B (RhB) molecules within MOF’s cavities, we followed a trivial postsynthetic method in which 30 mg of MOF-808 were added to an aqueous solution of RhB (1 mmol) and stirred overnight. The obtained mixture was centrifuged 5 times at 1000 rpm for 10 min and washed with additional water. The collected magenta powder, named hereafter RhB–MOF-808, was dried in ambient air. Evidence supporting the successful encapsulation of the dye within the MOF cavities is provided by the decrease in the Brunauer–Emmett–Teller (BET) surface area of the loaded sample. Indeed, through N_2_ adsorption measurements, we verified that the internal surface area was 577 m^2^/g, approximately four times lower than that of the pristine MOF.^21^ We assessed the integrity of the MOF by acquiring XRD patterns before and after dye encapsulation (see Figure S1c in the SI). No changes in peak positions were observed; thus, it can be concluded that the crystalline structure of the MOF remained unchanged.

We loaded Cou molecules into the MOF-808 matrix using the same postsynthetic method applied for RhB while using ethanol (EtOH) as the solvent, considering coumarin’s higher solubility in it.^39^ The resulting loaded powder is named hereafter Cou–MOF-808. For the random lasing experiment in aqueous solution, a 500 μL volume of aqueous RhB (1 mmol) was combined with a 500 μL volume of water in which MOF nanoparticles had been preliminarily suspended in variable concentrations. We decided to consider 4 different values to optimize scatterers’ concentration: Low (1.0 mg/mL), Medium (1.5 mg/mL), High (5.0 mg/mL), and Extra High (10.0 mg/mL). The same procedure, substituting water with ethanol, was used to prepare an aqueous suspension of Cou and MOFs in variable concentrations. We also prepared thin films composed of PVA embedding RhB (PVA RhB films) or RhB and MOFs (PVA RhB+MOF film). To this end, two separate procedures were followed. In the first, 47.9 mg of rhodamine B powder were dissolved in 10 mL of an aqueous PVA solution to prepare the bare PVA RhB film. In the second, 47.9 mg of rhodamine B powder and 20 mg of MOF-808 nanoparticles were added to 10 mL of the same aqueous PVA solution, yielding the PVA RhB+MOF film.

The resulting mixtures were then deposited on a Petri plate to obtain PVA RhB and PVA RhB+MOF films, which were then left to dry overnight and collected for further experiments.

### Optical Absorption

Optical absorption spectra were collected using an Avantes Star Line ULS2048CLEVO optical fiber spectrometer that worked in the 200–1300 nm range and was equipped with a dual Deuterium/Halogen lamp. Samples were placed in a 1 cm quartz cuvette.

### Steady-State and Time-Resolved Photoluminescence

Steady-state and time-resolved measurements were recorded by using an intensified charge-coupled device (CCD) camera. A wide range of excitation wavelengths was covered by a tunable laser system composed of an optical parametric oscillator (OPO) pumped by a Q-switched Nd:YAG laser with 5 ns pulses at a repetition rate of 10 Hz, with 0.1 mJ of energy. It enables the acquisition of steady-state photoluminescence spectra, imposing a temporal acquisition window that spans from before the impinging pulse’s arrival to a few milliseconds after the photoexcitation. On the other hand, it is possible to perform time-resolved measurements when the integrating window is shortened to 0.5 ns while continuously changing the delay with the laser pulse. The presented decay kinetics were extrapolated by the spectrally integrated emission intensity plotted as a function of time. By performing a least-squares fitting to a single exponential form (*I*(*t*) ∝ *e* ^–*t*/τ^) on the decay kinetics, it is possible to estimate the lifetime τ with an accuracy of 0.5 ns.

### Absolute Quantum Yield (QY) Measurements

The absolute photoluminescence quantum yields (QYs) of dyes and dyes–MOF nanocomposites have been estimated in an integrating sphere following standard protocols in the literature.^47^

The dye dispersions and the nanocomposite powder were inserted in quartz NMR tubes, positioned inside a LabSphere integrating sphere, and illuminated in indirect excitation geometry via a Thorlabs CW laser diode at 405 nm. The integrating sphere was coupled to an optical fiber spectrometer, enabling the acquisition of the spectral distribution of the emission (PL) and unabsorbed laser light (L) intensities.

Identical measurements were then performed on pure solvent and bare MOF powder as a reference in order to obtain the full incident laser intensity (L0). The QY was finally obtained as η = 100 × (PL)/(L0–L).

### Powder X-ray Diffraction (PXRD)

MOF-808 powder was put inside glass capillaries in order to collect PXRD measurements with a STOE STADI P machine set in Debye–Scherrer mode using Mo kα1 radiation.

### Scanning Electron Microscopy (SEM) Imaging

To perform SEM imaging, we utilized a JEOL JSM-IT800 microscope, while the MOF nanoparticles were dispersed in ethanol and drop-cast on copper tape.

### Random Lasing (RL) experiments

In the RL experiments, a solution of RhB (1 mmol, in water) or Cou (1 mmol, in ethanol) was used as a gain medium, in which we added MOF-808 nanoparticles (*d* ≈ 150 nm) as scatterers in variable concentrations. The source of the excitation pulses was a tunable laser, the irradiation system, a tunable laser, an OPOTEK VIBRANT (5 ns, 10 Hz). The beam passed through an aperture of 4 mm before being focused by a plano-convex cylindrical lens (*f* = 50 mm) on the plane of the sample. The active medium, consisting in a solution of dye in H_2_O or EtOH, with the addition of MOF nanoparticles, was contained inside a cylindrical vial or triangular cuvette to avoid any kind of Fabry–Perot resonant phenomena. The same setup was used to conduct RL experiments on Cou dye dissolved in ethanol as well. RL experiments were also conducted on powdered samples. In this case, a laser beam was focused on RhB–MOF-808 and Cou–MOF-808 powders by a 20× long working distance Olympus objective on the sample plane. The powder was held between two transparent glass slides. To avoid any scattering contributions from the excitation beam, we used, when necessary, a filter to block the excitation wavelength placed in front of the detector.

PVA RhB and PVA RhB+MOF films were mounted on a sample holder and positioned slightly away from the focal plane of the impinging pulsed pump. The light emitted from the active medium was collected at different pump energies (from 0.2 to 2.2 mJ/pulse) using an optical fiber (NA = 0.22) that led to a detection system composed of a monochromator (0.2 nm of spectral resolution) dispersing over a CCD camera synchronized with the pump source.

## Results and Discussion

In Figure 1e, we compare the photoluminescence (PL) of bare RhB in aqueous solution (1 mmol), excited by 552 nm radiation (2.2 mJ/pulse), with the emission centered at 596.8 nm, obtained from a solution of the same concentration enriched with MOF nanoparticles (1.5 mg/mL, size of ∼150 nm Figure S1a, superficial area ∼2090 m^2^/g).^21^ A full width at half-maximum (FWHM) of 34.8 nm was observed for bare RhB, which becomes approximately three times narrower (10.5 nm) when MOF scatterers are present in solution.

This clearly indicates the capabilities of MOF nanoparticles to enable random laser action, behaving as light scatterers when combined with the dye in the solution phase. In our random lasing experiments, we excited with a 552 nm wavelength, avoiding the possibility of stimulating an emission from the MOF nanoparticles themselves. In fact, in our previous work, we studied the optical properties of bare MOF-808 in aqueous solution, which can be photoexcited only in the UV range producing an emission peaking at 423 nm (Figure S2).^21^ Exciting in the visible guarantees that we populate only the excited states of RhB molecules, while the dispersed MOFs in solution solely act as scatterers. The photograph displayed in the inset of Figure 1e shows the emission from RhB molecules in the presence of MOF scatterers. The emission is omnidirectional, which is another characteristic signature of RL phenomena from a cylindrical geometry.^25^

Encouraged by the results obtained by mixing RhB and MOF-808 in solution, we proceeded to load RhB into the MOF-808 matrix, following the postsynthetic method previously discussed, in order to test the optical response of the resulting composite in the solid state. In general, it is well-known that aggregation-caused quenching phenomena prevent these highly emissive dyes from emitting.^12,45^ Here, we find that it is relatively easy to observe luminescence from RhB molecules when they are embedded within the MOF cavities, which keeps them sufficiently detached to avoid quenching.^8,9^ In fact, Figure 1g shows that RhB in the solid state is completely unable to glow under UV light (Figure 1g-i), in contrast to RhB–MOF-808, which appears highly emissive (Figure 1g-ii). Similar results are also observed under ordinary daylight (Figure 1g-iii,g-iv). As a matter of fact, RhB–MOF-808 emission is efficient enough to allow laser amplification when the pump energy is above a certain threshold (as analyzed later in the paper). In fact, the quantum yield (QY) of RhB–MOF-808 nanocomposites reaches (44 ± 1) %. Evidence of this is provided in Figure 1f, where we compare the emission collected from RhB–MOF-808 powders pumped by a 552 nm laser pulse focused through a 20× objective, at two different pumping intensities (0.5 and 2.2 mJ). At the highest power, we observe a substantial change in the shape of the emission, leading to a narrow random lasing peak centered at 606 nm with an FWHM of 11.6 nm. Thus, combining the dye with MOFs enables RL emission both in the solution phase (Figure 1e) and in the solid-state composite (Figure 1f).

Furthermore, considering MOFs’ extraordinary adhesive properties, related to their unparalleled value of internal surface area (∼2000 m^2^/g), their combination with the bright luminescence of guest dyes can be exploited for fingerprint pattern detection.^22^ In Figure 1h, we describe, as an example, pictures that show our attempts to detect a fingerprint previously imprinted over a glass slide: in the case of bare dyes (Cou is reported in Figure 1h-i; see RhB comparison Figure S3e,f), the pattern was not precisely reproduced, due to aggregation-induced PL quenching effects as mentioned earlier. Nevertheless, using dye-loaded MOF powder (Figure 1h-iii), we were able to trace the fingerprint pattern, which became more evident when the glass slides were exposed to UV light. To test the possibility of collecting fingerprints using the dye-loaded MOF powders with better resolution, we traced the intensity profile from pictures shown in Figure 1h-i,h-iii (see Figure S3 in the SI). Considering the intensity profile traced along a line crossing the fingerprint (Figure S3c), we evaluated the average difference between the peaks and the valleys of the intensity profiles: when using Cou–MOF-808 to impress the fingerprint, the intensity variation increased up to 30% with respect to the bare dye. Moreover, in the latter case, the intensity profile showed 30% fewer peaks compared to the dye-loaded MOF powder, confirming that a reliable and detailed fingerprint reproduction can be obtained. An alternative way to highlight a more detailed structure is to perform a Fourier transform (FT) on the image, which enables the decomposition of spatial domain information into its constituent frequency components. This transformation reveals specific patterns by converting spatial variations in pixel intensity into sinusoidal frequency components characterized by amplitude and phase, thereby facilitating the identification of periodicities, orientations, and spatial scales.^48,49^

Indeed, performing the FT of the same portion of the fingerprint images clearly reveals a structure only in the case of the fingerprint impressed by the Cou–MOF-808 composite, as evidenced by the comparison in Figure 1h-ii,h-iv.^48^

To further elucidate the emission properties of the RhB–MOF-808 and Cou–MOF-808 composite systems, we measured their emission quantum yields (QYs) and compared them to those of the respective free dyes. The RhB–MOF composite exhibited a QY of 44%, compared to 76% for bare RhB in H_2_O, while Cou–MOF-808 showed a QY of 6%, relative to 43% for the dye dispersed in EtOH. Although a decrease in the quantum yield (QY) of the encapsulated dyes compared to the free dyes is observed, it is important to emphasize that the incorporation of the chromophores within the MOF matrix yields solid-state emissive systems, which are potentially attractive for device-oriented applications. Another advantage provided by the hosting platform is enhanced photostability. To investigate this phenomenon, we monitored in situ the relative variation in emission intensity of the free dyes excited by a pulsed UV source (10 Hz) on the nanosecond time scale (see Figure S4a,b in the SI). Over the analyzed interval of 50,000 pulses, the stability of RhB increased by a factor greater than five when embedded within the MOF cavities, while Coumarin exhibited no bleaching effects when hosted in the MOF pores.

To better characterize the random lasing response of the dye-loaded MOF solution-phase mixture, we studied their emission as a function of laser power and scatterer concentration. In Figure 2a, we reported intensities and FWHM emitted from a 1 mmol solution of bare RhB as a function of pumping power: up to a low power value of 0.7 mJ/pulse (see also normalized spectra in Figure 2f), the emission band remains very broad (spontaneous emission), with FWHM = 50.1 nm. Above 1.12 mJ pumping power, the spontaneous emission gets amplified, and we observe a moderate narrowing down to 20.2 nm FWHM, considering the peak at higher energies, which remains substantially broader than the lasing spectrum in Figure 1a and still dominates by fluorescence. In contrast, the addition of MOFs in solution at a concentration of 1.0 mg/mL strongly enhances the narrowing effect, allowing the generation of random lasing above a certain power threshold (1.06 mJ/pulse). This is clearly demonstrated by Figure 2g, in which a series of normalized spectra of RhB+MOFs as a function of power are reported.

**Figure 2:**
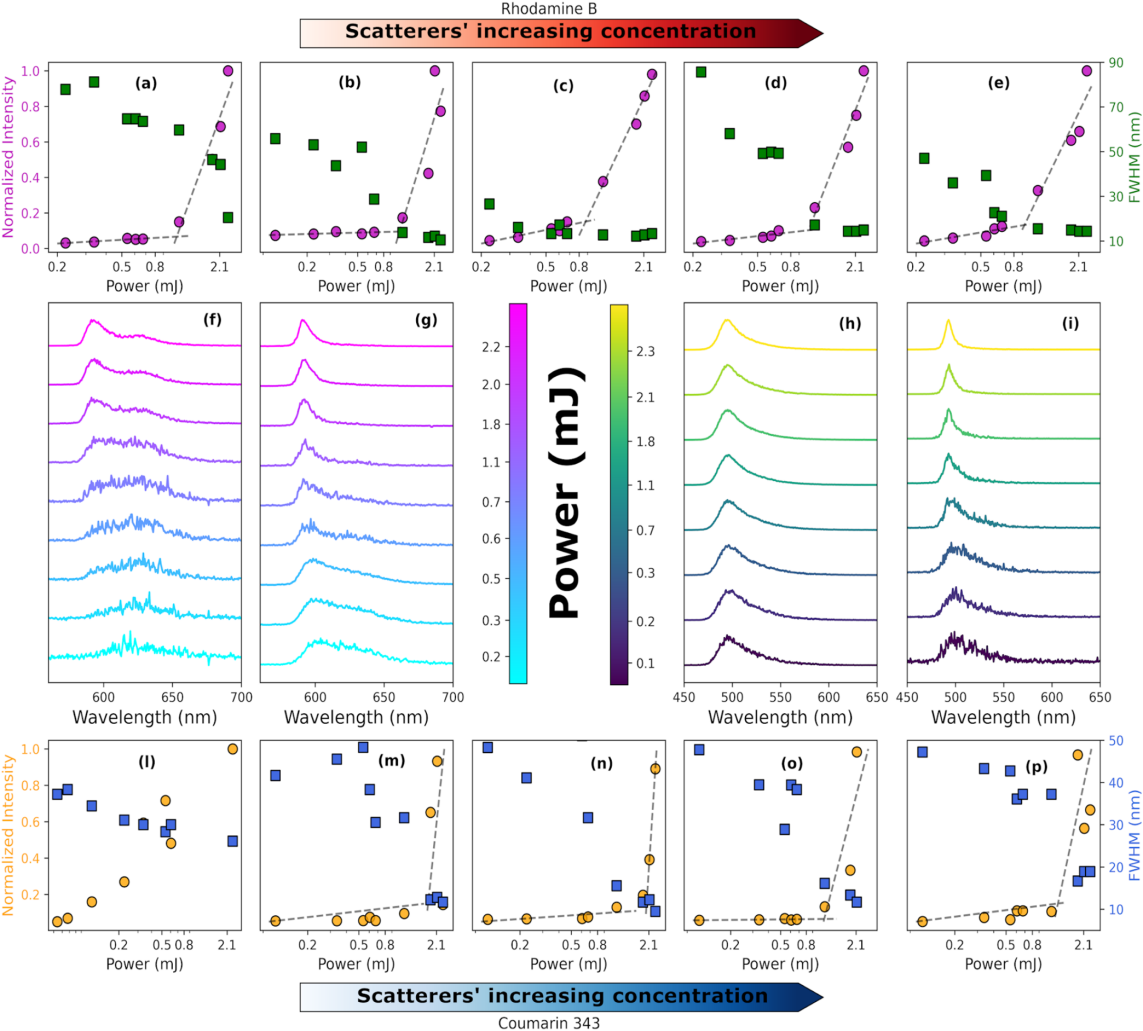
(a–e) Threshold plots of solutions of RhB to which was progressively added an increasing concentration of scatterers starting from 0 mg/mL (bare RhB, panel a), Low (1.0 mg/mL, panel b), Medium (1.5 mg/mL, panel c), High (5.0 mg/mL, panel d), and Extra High (10.0 mg/mL, panel e). (f) Normalized spectra of bare RhB as a function of power. (g) Normalized spectra of RhB with the addition of MOFs (1.0 mg/mL) as a function of power. (h) Normalized spectra of Cou as a function of power. (i) Normalized spectra of Cou with the addition of MOFs (1.5 mg/mL) as a function of power. (l–p) Threshold plots of solutions of Cou in EtOH, in the presence of MOF nanoparticles with the same progression of increasing concentrations used for RhB in panels a–e.

It is evident that bare RhB, even at the highest power value, maintains its fluorescence. Moreover, we also studied the lasing behavior of RhB as a function of scatterer concentration. Starting from a concentration of 1.0 mg/mL, named hereafter “Low” (Figure 2b,2g), we increased the concentration to 1.5 mg/mL (“Medium”, Figure 2c), 5 mg/mL (“High”, Figure 2d), and 10 mg/mL (“Extra High”, Figure 2e). At Low concentration, we estimated a lasing threshold at 1.06 mJ, which produces an FWHM of 10.6 nm (Figure 2b,g). The threshold reaches its minimum value (0.88 mJ) for the Medium concentration (Figure 2c), which also yields a minimum FWHM of 10.1 nm, while further increasing scatterer concentration produces an increase in the lasing threshold and a broadening of the band (13.9 nm for High concentration and 14.1 nm for Extra High concentration).

We repeated the same procedure with Cou dye dispersed in ethanol. For the bare dye, as shown in Figure 2l, intensity and FWHM values are reported as a function of pump power (pump wavelength fixed at 420 nm). Bare Cou does not show any lasing behavior even at high power; in fact, the lowest FWHM value is 26.2 nm (Figure 2l,h), which is similar to the one estimated at low power. The addition of MOF nanoparticles allows the switch-on of a lasing emission above a certain threshold (Figure 2i).

Normalized spectra of bare Cou and Cou+MOFs at Medium concentration (same concentration as in the RhB case) as a function of power are shown in Figure 2h,i. In the case of pristine Cou, the position of the fluorescence peaks remains unchanged as a function of power (495.1 nm), and the variations of FWHM are very small (from 37.2 to 26.2 nm), while in the MOF-enriched solution, a clear narrower peak arises at 493.1 nm, reaching an FWHM of 9.3 nm at Medium concentration above the lasing threshold.

Also, for Cou, the threshold value has been studied as a function of scatterer concentration. Low MOF concentration shows a threshold at 1.71 mJ accompanied by an FWHM of 11.5 nm (Figure 2m). Increasing the MOF concentration (1.5 mg/mL) leads to a further narrowing of the peak, reaching an FWHM of 9.3 nm, even if the threshold increases up to 1.89 mJ for Medium concentration (Figure 2n). We added MOF nanoparticles also reaching High and Extra High concentrations, recording comparable threshold values of 1.15–1.21 mJ, lower with respect to the previous concentrations, while we measured FWHM values of 11.5 and 19.7 nm, respectively. Upon examination of Tables S1 and S2 reported in the SI, which report the lasing threshold and FWHM values, a distinct minimum in the FWHM trend is observed for both rhodamine B and coumarin 343 at an intermediate scatterer concentration of 1.5 mg/mL. In interpreting this behavior, the FWHM values are considered more reliable due to the high spectral resolution achieved (0.2 nm), whereas the threshold estimates are subject to greater relative uncertainty owing to multiple sources of experimental error.

The emergence of a minimum FWHM of 1.5 mg/mL may suggest the existence of an optimal scatterer concentration that enhances light scattering within the gain medium. Beyond this concentration, an increased tendency for nanoparticle agglomeration in the liquid phase may lead to accelerated precipitation, reducing the effective number of scatterers in the system. This loss negatively impacts the lasing process, as reflected in the marked broadening of the emission spectra observed at higher concentrations for both dyes.

Finally, by comparing RhB and Cou (Tables S1 and S2) behaviors as a function of power and scatterer concentration, it is evident that RhB works better as an active medium for random lasing, producing a narrower band (10.1 nm) at lower power (0.8 mJ) at Medium scatterer concentration. It is reasonable that the RhB dye works better than the Cou dye, considering the QY values of dye–MOF nanocomposites, resulting in ∼44% and ∼6% for RhB–MOF-808 and Cou–MOF-808, respectively.

To correlate the scatterers’ density with threshold values, we evaluated the changes in the scattering mean free path (MFP) for different concentrations of MOF-808 nanoparticles. We estimated the MFP through the following equation^50^

$$\mathrm{MFP}=\frac{T}{\ln\left(\frac{I_{1}}{I_{2}}\right)}$$

where *T* is the thickness of the sample (quartz cuvette, 1 mm path length), *I*_1_ is the transmitted intensity through the pure solvent (water for RhB, while EtOH in the case of Cou), and *I*_2_ is the transmitted intensity of the solvent containing scatterers in a known concentration. The estimated values of MFP as a function of wavelength are reported in Figure S5 in the SI; for three different scatterer concentrations, the values resulted in the range of 0.5–3.0 mm, and the trend is downward as concentration increased. It is known that in the case of coherent feedback, MFP values are comparable with the emission wavelength, which is typical of a strongly scattering medium. Since in our case, photons’ mean free path largely exceeds the emission wavelength of the utilized dyes, the RL systems are expected to show incoherent feedback typical for weakly scattering regimes.^22^ We estimated the value of *l*_c_, corresponding to the optical cavity length, by performing the power Fourier transform (PFT) of the emission spectra of the RhB–MOF-808 system and the corresponding liquid-phase system, in order to gain further insights into the lasing modes.^51,52^ Using the PFT of the emission spectra reported in Figure S6 in the SI, we calculated the resonator sizes, obtaining values of 25 μm for *l*_c_ in the liquid-phase system and 30 μm for the solid-state system. These results are consistent with the hypothesis of an incoherent regime, as the cavity length is significantly larger than the emission wavelength.^22^

A higher scatterer density should lead to an increase in scattering events, and hence, the confinement of photons within the active volume and amplification increase, resulting in a decreased threshold.

Besides studying the dependence on excitation intensity, an alternative way to verify the onset of a lasing phenomenon is to compare the decay kinetics of the lasing peak with that of the spontaneous emission. For this reason, we performed time-resolved fluorescence spectroscopy on a bare RhB solution, shown in Figure 3a as a contour map, where it is possible to monitor intensity changes as a function of time delays relative to different emission wavelengths. The decay kinetics of bare RhB is well fitted by a single exponential function, and the estimated lifetime is 1.4 ns. The same measurements were repeated on RhB+MOF-808 solution at the 1.5 mg/mL concentration of scatterers (Figure 3b). The peak maintains its character along the entire experiment, while appearing narrower with respect to the bare dye (as shown in Figure 2f,g), and the decay kinetics show a lifetime below 1 ns, resulting in pulse-limited behavior by our time resolution (compared with the pump’s decay kinetics). Both kinetics are reported and compared in the inset of Figure 3b. The reduction of the lifetime in the presence of MOF scatterers is another clear indication of the onset of laser amplification. In Figure S7a, we report a more systematic study of the decay kinetics as a function of the pump power for the RhB+MOF-808 solution (1.5 mg/mL): the kinetics corresponding to power values from 1.8 mJ/pulse to 0.3 mJ/pulse showed a good degree of superposition, even if 0.3 mJ/pulse produces a longer lifetime with respect to higher energy power, with an initial component with a steeper slope (up to 3 ns of time delay, pulse-limited), followed by decay kinetics similar to bare RhB. The kinetics collected with a pump power of 0.2 mJ/pulse and below almost overlaps with the decay kinetics of RhB (Figure S7b). This allows for estimating the lasing threshold to be above 0.3 mJ/pulse through a comparison of the decay kinetics.

**Figure 3:**
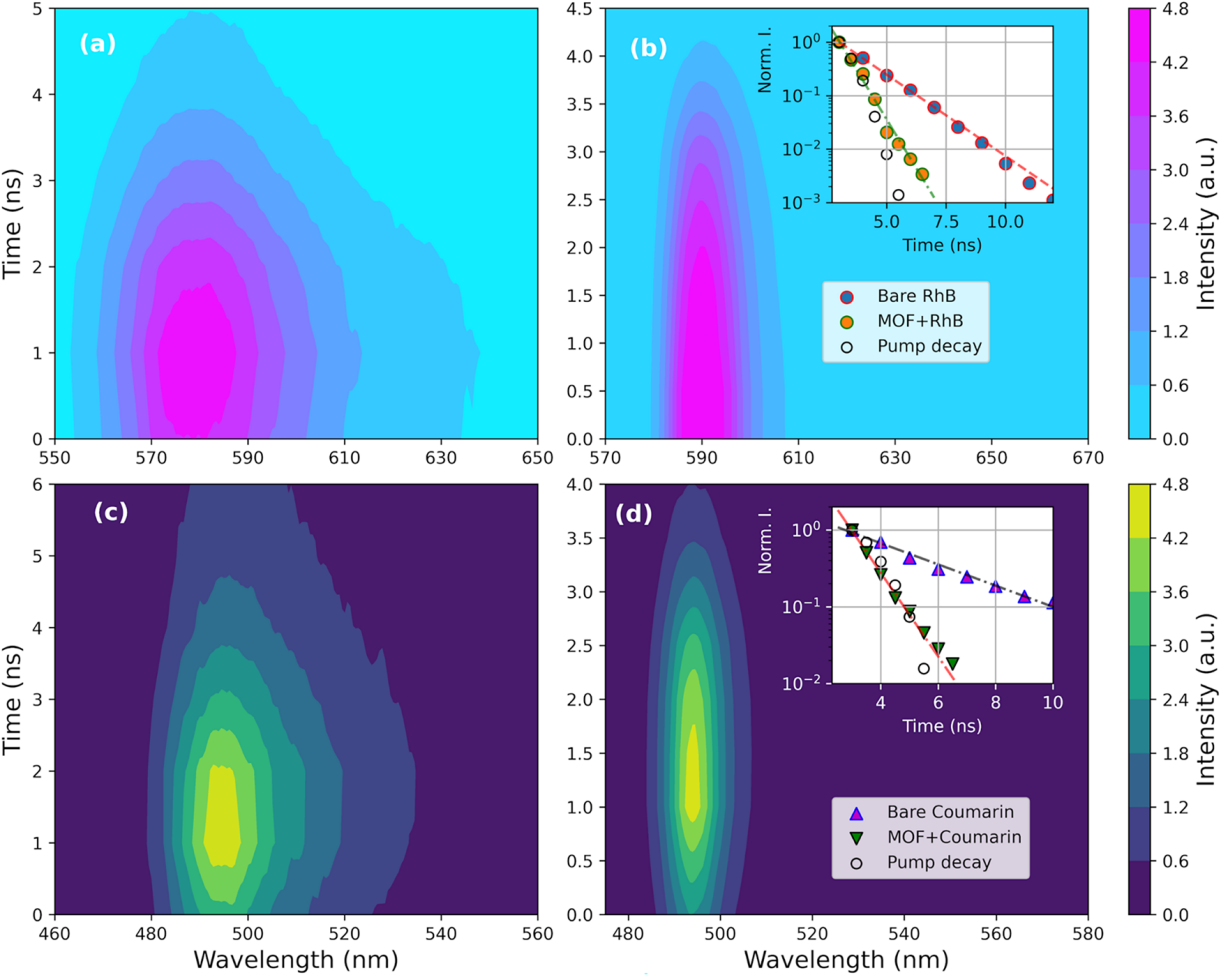
Contour plot of emission spectra collected as a function of delay time from excitation laser pulse in (a) aqueous solution of RhB; (b) RhB with MOF nanoparticles, 1.5 mg/mL; (c) Cou dissolved in EtOH; and (d) Cou solution where MOF nanoparticles were added (1.5 mg/mL). The intensity values are referred to the scale bar on the right. Kinetic decays derived from (a) and (b), (c) and (d), respectively, are shown in the inset and compared.

We extended time-resolved experiments also to Cou dissolved in EtOH, and its intensity as a function of time is reported in Figure 3c, while the corresponding decay kinetics is displayed in the inset of Figure 3d; the trend is well fitted by a single exponential with an estimated lifetime of 3.2 ns. Kinetics recorded on the Cou solution where scatterers were added at a 1.5 mg/mL concentration is shown in Figure 3d, where it is possible to verify the characteristic narrowing. Similar to RhB, the relative decay kinetics is faster than that of the bare dye, and the lifetime turns out to be limited by our time resolution (as shown in the inset of Figure 4d). In Figure S8 in the SI, we reported the kinetic decays of the Cou+MOF-808 at Medium concentration as a function of pumping power. At all of the energy values above threshold, the traces resulted in identical behavior, and the lifetime is faster (while resolution-limited) than the lifetime recorded at low power (below threshold), which showed a superimposition with one of bare Cou (3.2 ns).^53^

**Figure 4:**
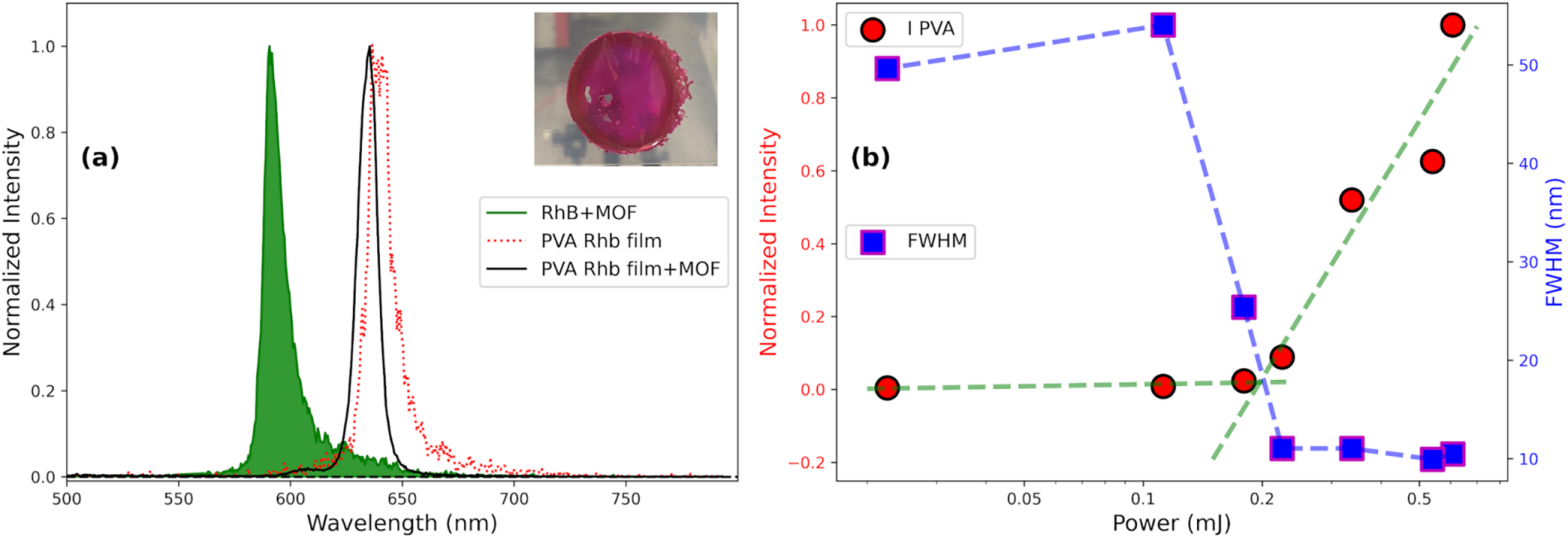
(a) Normalized lasing spectra of RhB in aqueous solution with the addition of MOF scatterers at Low concentration (1.0 mg/mL, filled green curve), polymeric film prepared starting from a solution of bare RhB (red dotted curve), while the continuous black curve represents the spectrum of the film obtained with the addition of MOF nanoparticles as scatterers (shown in the inset as a picture). (b) Threshold plot of the polymeric film containing MOF nanoparticles (10 mg/mL), in which red dots represent the intensity as a function of increasing power, while blue squares represent the FWHM. Dashed lines are a guide to the eye.

Finally, we also investigated the impact of scatterers’ concentration on the kinetic decay properties. In Figure S9, the kinetic decays of RhB and Cou in the presence of MOF-808 nanoparticles at different concentrations are reported. Both solutions were excited by 2.2 mJ pumping pulses to take the kinetic traces above the lasing threshold. The kinetic traces showed a good degree of superposition, suggesting the independence of the decays with respect to concentration, at least when the impinging pumping pulse has an energy above the estimated threshold.

By leveraging the information obtained from the concentration optimization study, we tested the feasibility and potential gains of incorporating MOF-808 nanoparticles into a portable device. We attempted to observe lasing occurring from a solid macroscopic system, that is, a portable polymeric thin film (Figure 4a, inset). To prepare the film, we started from a solution of poly­(vinyl alcohol) (PVA) in water (2 mmol) to which we added RhB to reach a 1 mmol concentration and 20 mg of MOF nanoparticles. The obtained viscous solution was deposited on a Petri plate and then ambient dried. The main PL emission of RhB in PVA is red-shifted by 40 nm with respect to the RhB aqueous solution (Figure S10). Such an effect is probably due to the interaction between the dye’s molecules and the polymeric matrix.^43^ We first compared the narrowing effect and lasing threshold of a PVA film containing only RhB molecules with respect to RhB in water (Figure 4a).

In Figure 4a, we compare the peak recorded from aqueous RhB+MOFs with the peak of RhB in the PVA film with the addition of MOFs (centered at 635.4 nm, FWHM = 10.5 nm) and to the bare dye film peak (centered at 639.3 nm, FWHM = 11 nm). Substantial narrowing, comparable to the aqueous case, is observed in the PVA film independent of the presence of MOFs. To estimate the lasing threshold, it is possible to plot the recorded FWHM and intensity values as a function of pumping power (Figure 4b), as was already done for the solution phase in Figure 2. It is evident that after overcoming a certain value of power (above 0.2 mJ), intensity values start to rise, and simultaneously, FWHM starts to decrease. MOF nanoparticles do not have a remarkable effect on the lasing mode narrowing, as evident in Figure 4a, indicating that the environment, i.e., the PVA matrix, as water in the previous cases, is the ultimate cause of inhomogeneous broadening, which limits the width. On the other hand, MOFs dramatically affect the threshold: the threshold of the bare RhB film (0.6 mJ/pulses) is halved due to the presence of scatterers (see Figure S11a in the SI).

Indeed, the addition of MOF nanoparticles in the film is expected to favorably impact the value of the lasing threshold through a reduction of photons’ mean free path (MFP) in the gain medium, as reported by Chen and co-workers.^54^ To test the impact of concentration, we prepared a thin film embedding a lower concentration of MOF nanoparticles (5 mg/mL), which was found to display a higher lasing threshold of 0.6 mJ/pulse (see Figure S11b in the SI). We carried out time-resolved nanosecond analysis, finding that the kinetic decay of the RhB PVA film could be well fitted by a single exponential decay with a lifetime of 1.3 ns, suggesting a similarity with respect to bare RhB in aqueous solution.^53^ The film enriched with MOF nanoparticles (Figure S11c) showed a faster decay, which was time-resolution-limited by our setup. The lowering of the energy threshold, together with the lifetime shortening, clearly shows that MOFs make dye random lasing more efficient. The observation of lasing from thin films paves the way for novel applications, including the development of portable optoelectronic devices, such as solid-state lasers for biomarker detection.^55^

## Conclusions

We investigate the role of MOF-808 in the optical behavior of fluorescent dyes. Our spectroscopic results demonstrate that MOF-808 nanoparticles significantly enhance random lasing emission by acting as effective scatterers, reducing the FWHM of emission spectra and lowering lasing thresholds. We explore the dependence of the lasing behavior on the MOF concentration and excitation intensity. Increasing the MOF concentration initially reduces the lasing threshold and narrows emission spectra, but excessive concentrations result in increased thresholds and spectral broadening. In particular, our results demonstrate that MOF-808, hosting dye molecules in its cavities, prevents aggregation-induced quenching, allowing the use of dye powder and of their emission in solid-state applications such as dactyloscopy. Finally, we extend our investigation to polymeric thin films incorporating both dye molecules and MOF nanoparticles, demonstrating their feasibility for portable lasing applications with high performance, considering that MOFs drastically lower the lasing threshold by halving it compared to bare RhB films. Our findings establish MOF-808 nanoparticles as highly effective scatterers for random lasing applications, enabling efficient lasing emission in both solution and solid state, with promising implications for sensing and portable laser technologies.

## Supplementary Material



## Notes

SEM; XRD; time-resolved photoluminescence; and photostability tests (PDF)

## ⊥

Laboratoire des Solides Irradiés, CEA/DRF/lRAMIS, Ecole Polytechnique, CNRS, Institut Polytechnique de Paris, F-91128 Palaiseau, France
