# Supplementary material for: Host–Guest Engineering of MOF-808 for Random Lasing and Solid-State Emission
Source: ACS Appl Nano Mater. 2025 Jul 18;8(30):15165–75. doi: 10.1021/acsanm.5c02396 (PMC12322874; doi:10.1021/acsanm.5c02396)
Supplement: Supplementary file 1 [file an5c02396_si_001.pdf]

Supporting Information

Host–Guest Engineering of MOF-808 for Random Lasing and Solid-State Emission

Giuseppe Ficarra, [a] Ashim Pramanik, [a] Ludovico G. Barbata, [a] Marco Cannas, [a, b] Romy L. Ettlinger, [c,d] Russel E. Morris, [c] Gianpiero Buscarino,[,a] Fabrizio Messina, [*,a] and Alice Sciortino[*,a]*

[a] Physics and Chemistry Department - Emilio Segrè, University of Palermo, Via Archirafi 36, 90123, Palermo, Italy

[b] INST Consortium for Materials Science and Technology, Via Giusti 9, 50125, Firenze, Italy

[c] EastChem School of Chemistry, University of St Andrews, North Haugh, St Andrews, UK

[d] TUM School of Natural Sciences, Technical University of Munich Lichtenbergstr.4;85748

Email: gianpiero.buscarino@unipa.it, fabrizio.messina@unipa.it, alice.sciortino02@unipa.it

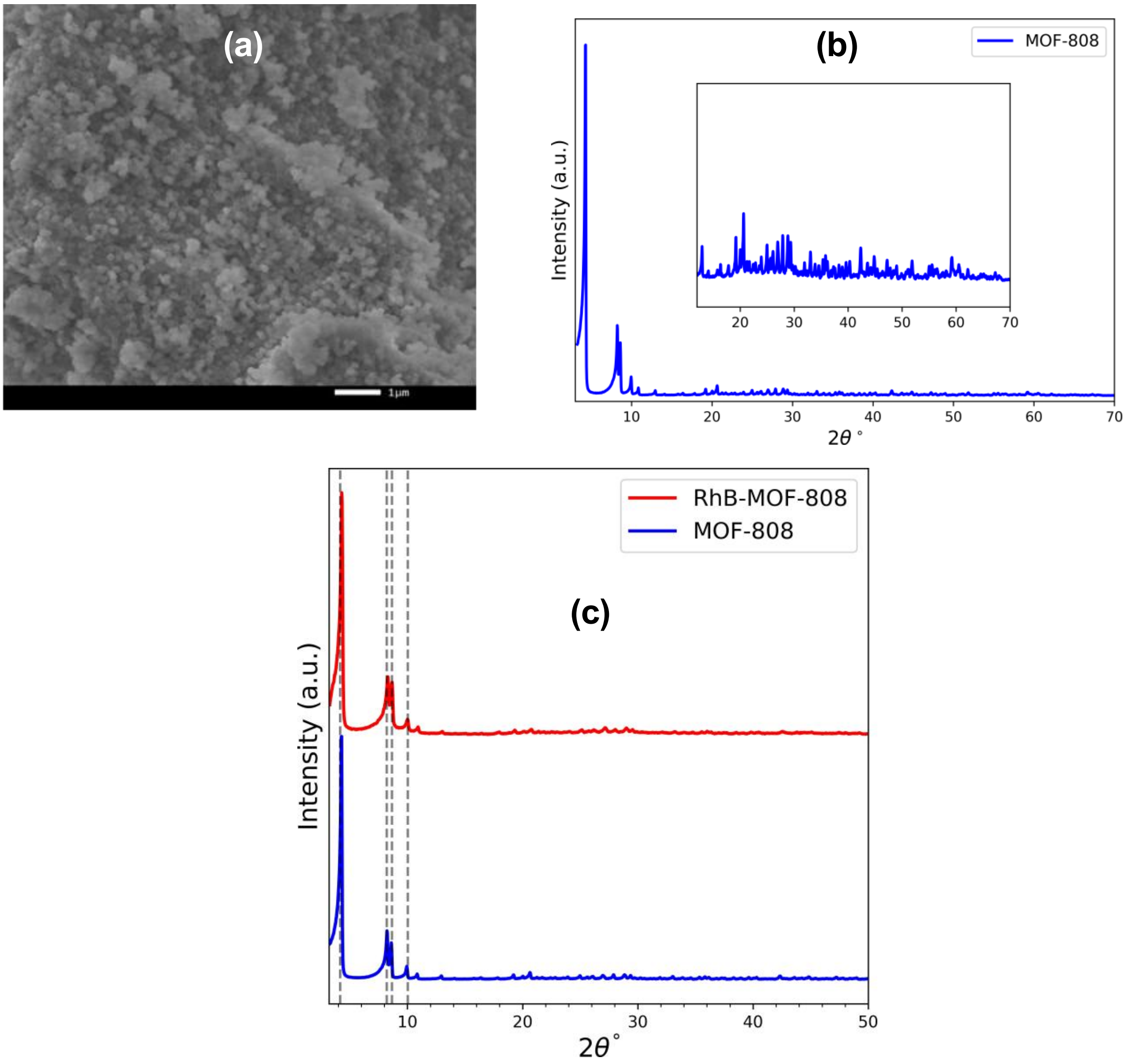

Figure S1 (a) SEM image of MOF-808 nanoparticles from which it is possible to estimate a mean size distribution with a mean value of 150 nm. **(b)** XRD pattern of MOF-808 with the inset displaying a zoom in the region from 12° to 70°. **(c)** XRD pattern of pristine MOF-808 (blue) compared with the XRD pattern of loaded RhB-MOF-808 (red). Dashed lines serve as a guide to the eye, highlighting the positions of the main peaks.

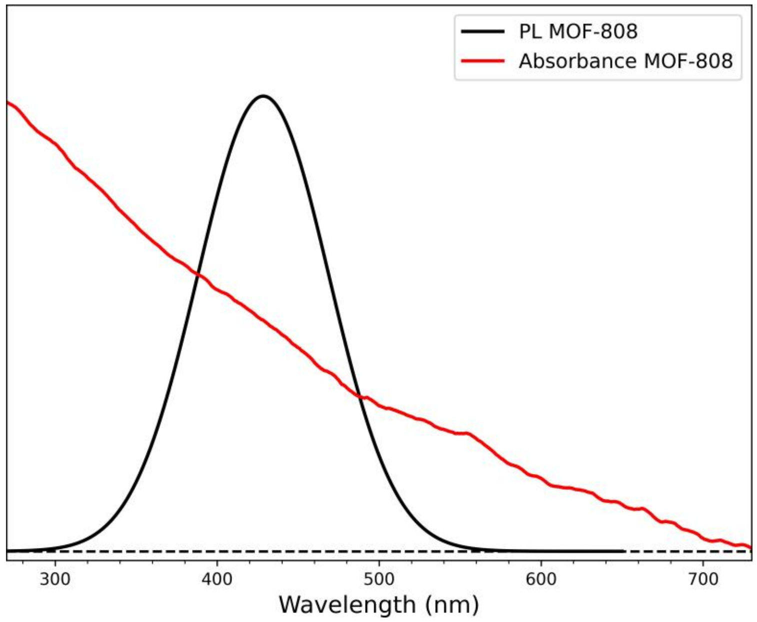

Figure S2 Emission spectrum of MOF-808 (black curve) collected after 280 nm excitation pulse. Absorbance spectrum is reported in red.

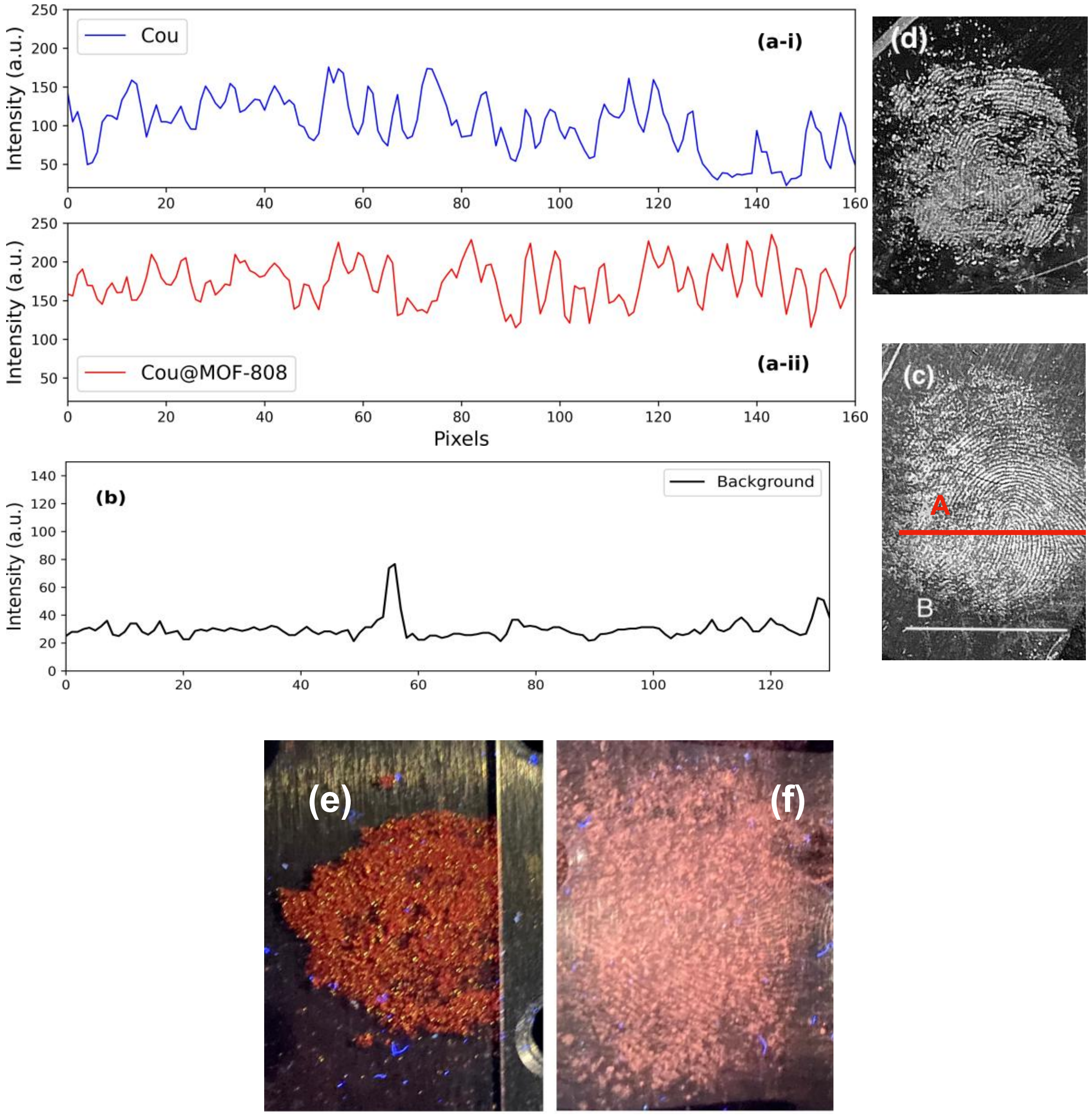

Figure S3 (a-i) Intensity profile from image **d** (fingerprint impressed on bare Coumarin powder) along line A. **(a-ii)** Intensity profile from image **c** (fingerprint impressed on Cou@MOF-808 powder). **(b)** Intensity profile as a function of pixels of the image **c** along line B. **(c)** Fingerprint impressed on Cou@MOF-808 powder. **(d)** Fingerprint impressed on bare Coumarin powder. **(e)** Fingerprint impressed on bare RhB powde**r** **(f)** Fingerprint impressed on RhB@MOF-808 powder. The showed fingerprints belong to the same person.

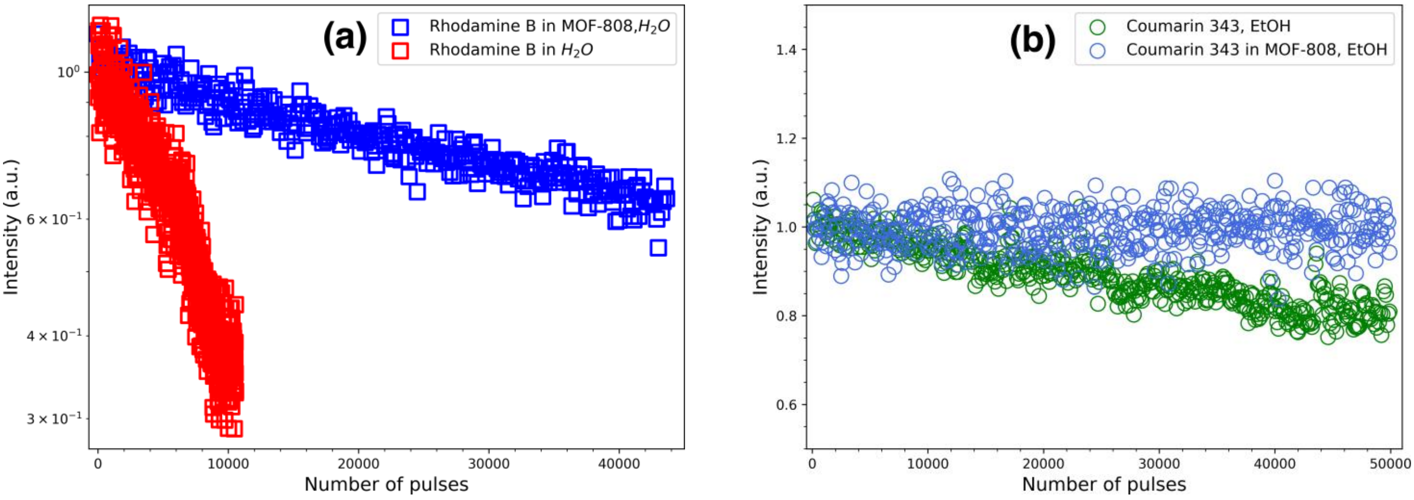

Figure S4 (a) Photobleaching kinetic of bare Rhodamine B in H_2O (red squares) and Rhodamine B encapsulated within MOF-808 cavities (blue squares). **(b)** Photobleaching kinetic of bare Coumarin 343 B in EtOH (green circles) and Couamrin 343 encapsulated within MOF-808 cavities (blue circles).

Rhodamine B	MOF-808 conc. (mg/ml)	Threshold (mJ/ pulse)	Minimun FWHM (nm)
	0	1.10	13.4
LOW	1	1.06	10.6
MEDIUM	1.5	0.88	10.1
HIGH	5	1.01	13.9
EXTRA HIGH	10	1.02	14.1

Table S1 Summary table containing the key results such as MOF's nanoparticles concentration (mg/ml), estimated threshold (mJ/pulse) and minimum registered FWHM (nm) in the case of Rhodamine B dissolved in H_2O.

Coumarin 343	MOF-808 conc. (mg/ml)	Threshold (mJ/ pulse)	Minimun FWHM (nm)
	0	Unattributed	26.2
LOW	1	1.71	11.5
MEDIUM	1.5	1.89	9.3
HIGH	5	1.15	11.5
EXTRA HIGH	10	1.21	19.7

Table S2 Summary table containing the key results such as MOF's nanoparticles concentration (mg/ml), estimated threshold (mJ/pulse) and minimum registered FWHM (nm) in the case of Coumarin 343 dissolved in Ethanol.

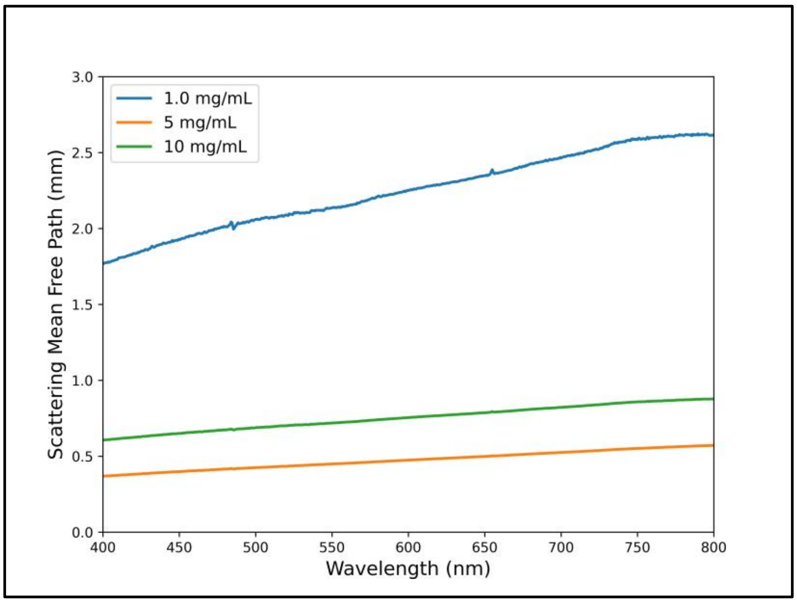

Figure S5 Mean free path (MFP) estimated for 3 different concentrations of MOF-808 nanoparticles in aqueous solution. MFP corresponding to each wavelength was extracted following the equation: $MFP = \frac{T}{ln(I_1 / I_2)}$, where T is the path length of the sample (1 mm quartz cuvette containing the solution) while I_1 and I_2 are referred to the intensities of transmittance spectra of the bare solvent and the solvent with a fixed concentration of scatterers.

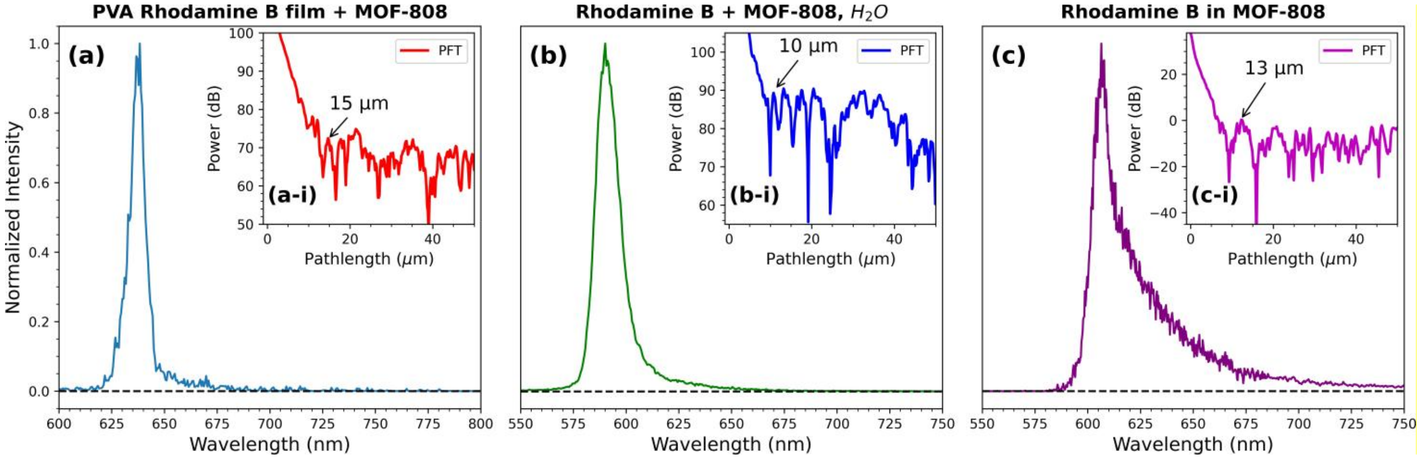

Figure S6 (a) Emission spectra of PVA Rhodamine B film with the addition of MOF-808 nanoparticles above the lasing threshold. **(a-i)** PFT of the spectrum in (a) in which is highlighted the first order peak corresponding to 15 μm of pathlength. **(b)** Emission spectra of Rhodamine B aqueous solution with the addition of MOF-808 nanoparticles (1.5 mg/ml) above the lasing threshold. **(b-i)** PFT of the spectrum in (b) in which is highlighted the first order peak corresponding to 10 μm of pathlength. **(c)** Emission spectra of Rhodamine B embedded in MOF-808; the powder was pumped with an energy above the lasing threshold. **(c-i)** PFT of the spectrum in (c) in which is highlighted the first order peak corresponding to 13 μm of pathlength.

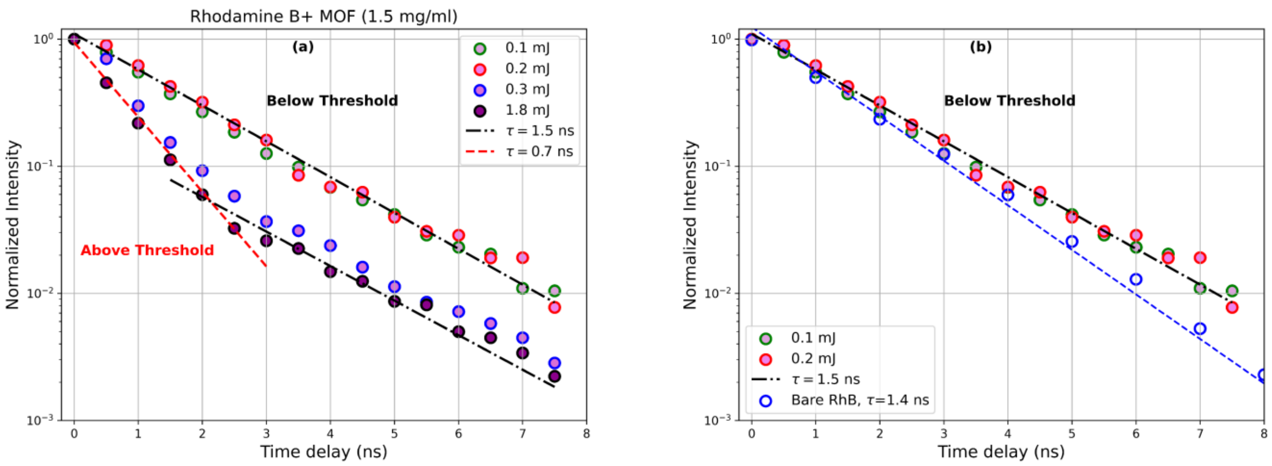

Figure S7 (a) Kinetic decays of Rhodamine B in water (1.5 mg/ml) at different power of the excitation pump pulse (552 nm). The traces corresponding to the above threshold powers (1.8-0.3 mJ/pulse) show an initial component with a steeper slope (up to 3 ns of time delay, pulse limited), followed by a decay kinetics more akin to that of the bare dye, =1.5 ns. **(b)** Kinetic decay of Rhodamine B+ MOF nanoparticles at low excitation intensities (1.5 mg/mL, 0.2 mJ excitation pulse) compared with the kinetic decay of bare RhB in water.

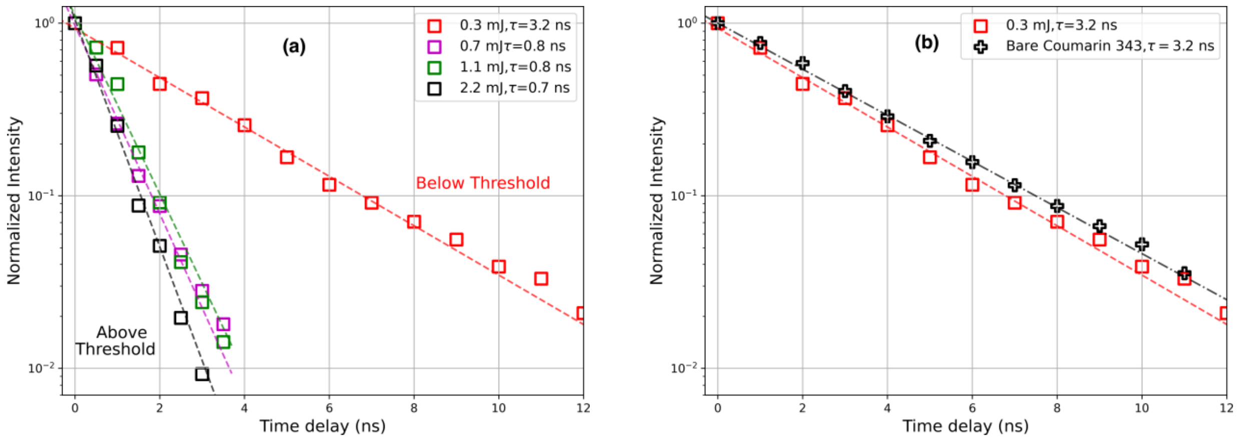

Figure S8 Kinetic decays of Coumarin 343 in EtOH in which where added MOFs' nanoparticles in 1.5 mg/mL concentration. The red squares represent the kinetic after 0.3 mJ excitation pulse (420 nm), which is fitted with a single exponential with 3.2 ns lifetime. The magenta, green and black squares are the kinetic decay registered when the excitation pulse power was set at 0.7, 1.1 and 2.2 mJ respectively. **(b)** Kinetic decay of Coumarin 343 + MOF nanoparticles (1.5 mg/mL, 0.3 mJ excitation pulse) compared with the kinetic decay of bare Coumarin 343 dye in Ethanol.

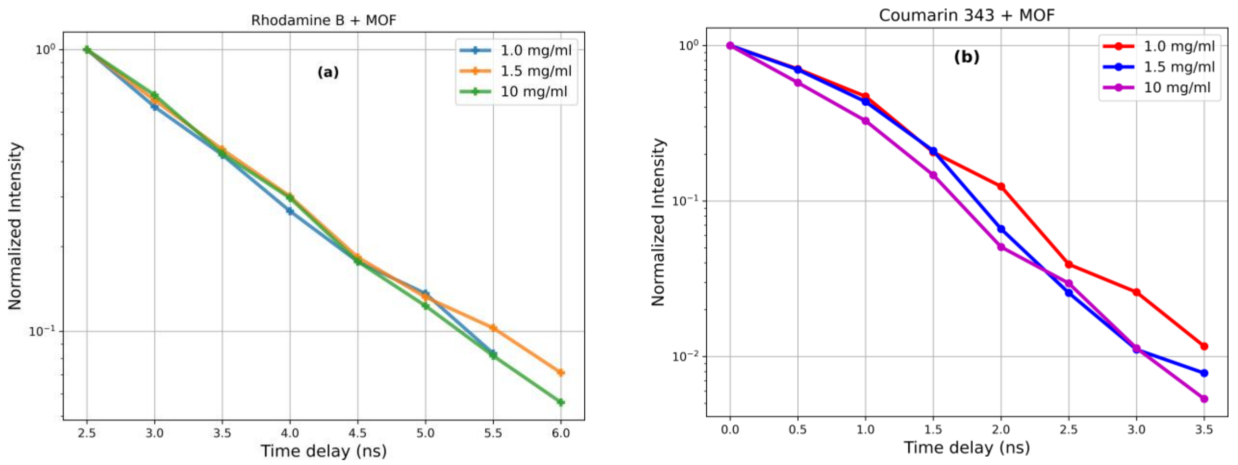

Figure S9 (a) Kinetic decays of RhB aqueous solution considering different scatterer concentrations, normalized after the first 2.5 ns of time delay. Regardless the scatterer concentration, the kinetic decay results the same (pumping pulse power is 2.2 mJ). **(b)** Kinetic decays of Coumarin 343 aqueous solution considering different scatterers' concentrationsNo matter what the scatterers' concentration was presents, the kinetic decay results the same (pumping pulse power is 2.2 mJ).

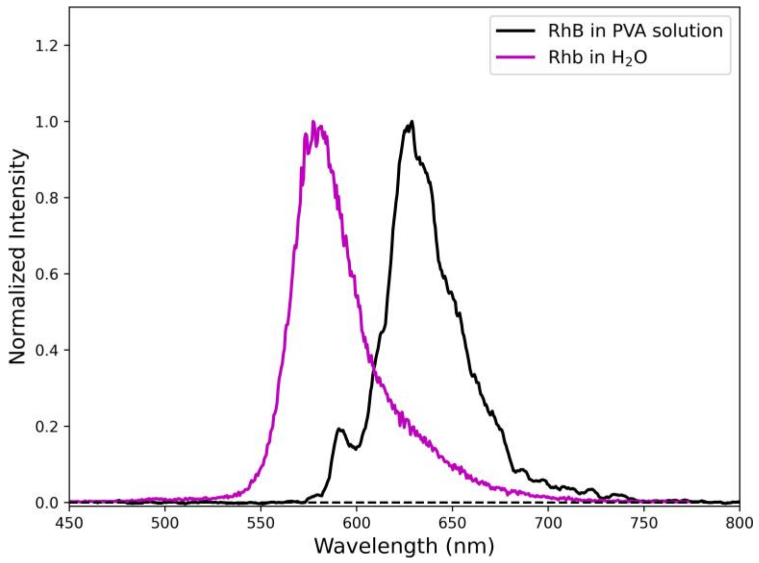

Figure S10 Luminescence spectrum of Rhodamine B in H_2O, excited at λ_{ex}= 552 nm and peaked at 590 nm (magenta curve) compared with the emission coming from a solution of PVA containing Rhodamine (black curve) excited with the same wavelength (peak at 630 nm). It is worth noticing that the black spectrum features a small peak at 590 nm, maybe due to the presence of non interacting Rhodamine B.

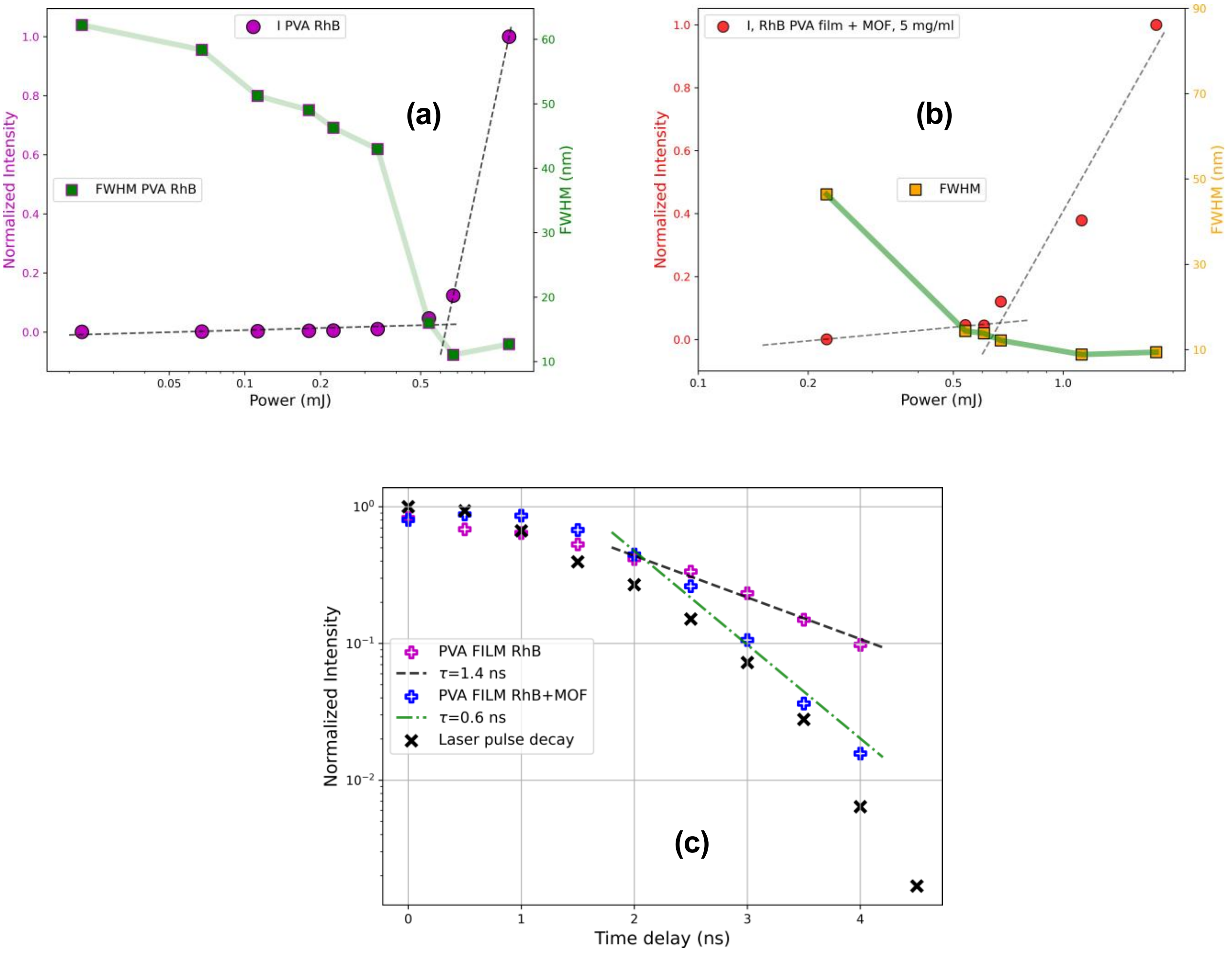

Figure S11 Threshold plot of PVA film consisting of bare RhB, estimated lasing threshold is 0.6 mJ: magenta dots are intensities values as a function of power, while green squares are the FWHM (green line is a guide to the eyes). **(b)** Threshold plot of PVA film prepared with the inclusion of MOF-808 nanoparticles (5mg/ml), estimated lasing threshold is 0.6 mJ: red dots are intensities values as a function of power, while yellow squares are the FWHM (green line is a guide to the eyes). **(c)** Kinetic decay of the thin film realized combining PVA and bare RhB (magenta points) in which the decreasing part of the kinetic has been fitted by a single exponential decay with a lifetime of 1.4 ns. Blue points are the kinetic decay of the thin film enriched with MOF-808 nanoparticles: the trend of the decay results faster with respect to the bare RhB film, reporting an estimated lifetime of 0.6 ns (pulse limited) as suggested by the superimposed laser's decay kinetic (black crosses).
